# From obscurity to opportunity: targeting *Neuregulin 1* fusions in solid tumors

**DOI:** 10.1038/s41392-025-02283-y

**Published:** 2025-06-27

**Authors:** Sofía Llorente, Angela Lamarca, Tian V. Tian

**Affiliations:** 1 Upper Gastrointestinal and Endocrine Tumor Unit, Vall d’Hebron Institute of Oncology, Vall d’Hebron University Hospital, Barcelona, Spain; 2 Department of Oncology, OncoHealth Institute, Instituto de Investigaciones Sanitarias FJD, Fundación Jiménez Díaz University Hospital, Madrid, Spain

**Keywords:** Oncology, Medical research

In a recent issue of *The New England Journal of Medicine*, an international team reported the final results of the Phase 2 eNRGy trial (NCT02912949), which evaluated the efficacy and safety of zenocutuzumab, a bispecific antibody against epidermal growth factor receptors 2 and 3 (HER2 and HER3), in patients with *Neuregulin 1 (NRG1)* fusion-positive solid tumors.^1^ The trial yielded compelling evidence of zenocutuzumab’s robust clinical efficacy in this molecularly defined population, accompanied by a favorable tolerability profile.

**Fig. 1:**
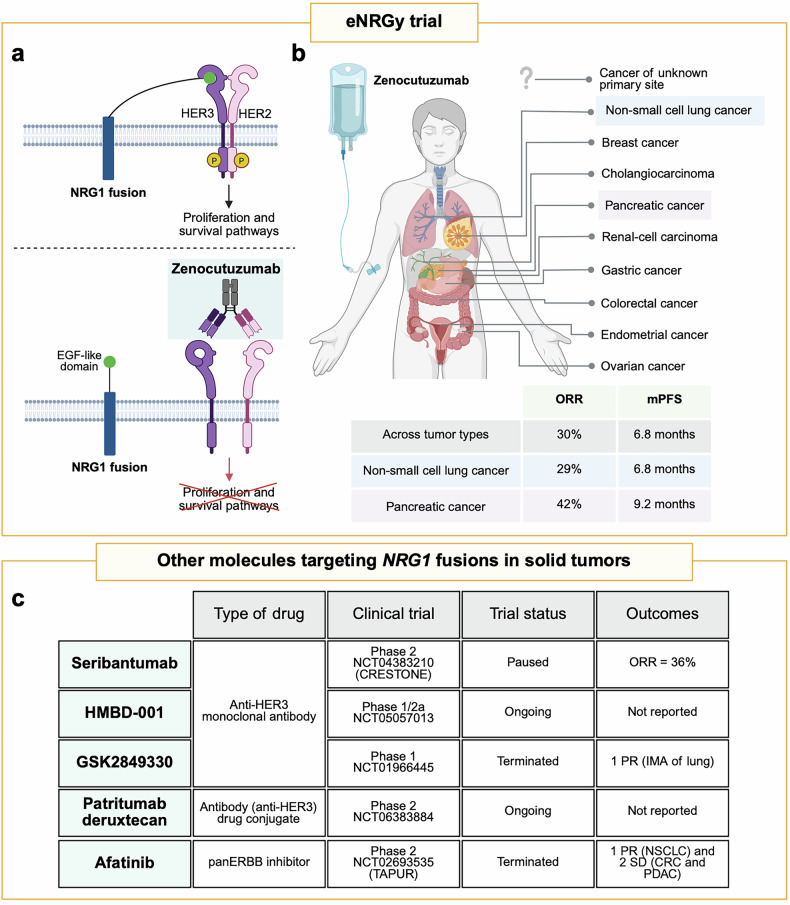
Graphical summary of the mechanism of action of zenocutuzumab, the main outcomes of the eNRGy trial, and other molecules targeting *NRG1* fusion-positive advanced solid tumors. **a** NRG1 fusion proteins bind to HER3 via their EGF-like domain, inducing HER3/HER2 heterodimerization and activating downstream signaling pathways involved in cell proliferation and survival. Zenocutuzumab is a bispecific antibody targeting both HER3 and HER2, thereby blocking NRG1 binding to HER3 and preventing the activation of cell proliferation and survival pathways. **b** In the eNRGy trial, patients with *NRG1* fusion-positive tumors, across 10 tumor types, were treated with zenocutuzumab. The study demonstrated impressive efficacy, particularly in NSCLC and pancreatic cancer. These encouraging results led to the FDA approval of zenocutuzumab as the first drug indicated for the treatment of *NRG1* fusion-positive NSCLC and pancreatic adenocarcinoma. **c** Table summarizing therapeutic agents targeting *NRG1* fusion-positive advanced solid tumors. NSCLC non-small cell lung cancer, IMA invasive mucinous adenocarcinoma, PDAC pancreatic ductal adenocarcinoma, CRC colorectal cancer, ORR objective response rate, mPFS median progression-free survival, PR partial response, SD stable disease. Figure generated using BioRender

Fusions involving the *NRG1* gene are rare in solid tumors, with an overall prevalence of less than 1% across various tumor types.^2^ However, they are notably enriched in specific malignancies, such as invasive mucinous adenocarcinomas (IMA), a subtype of non-small cell lung cancer (NSCLC), and in *KRAS* wild-type pancreatic ductal adenocarcinoma (PDAC). Unlike other driver fusions that form chimeric receptors, such as *NTRK*, *RET*, *ROS1*, *ALK*, and *FGFR* fusions, chimeric NRG1 fusion proteins function as ligands, driving oncogenic signaling by binding to HER3 through their epidermal growth factor (EGF)-like domain (Fig. 1a). This interaction can trigger HER2-HER3 heterodimerization, leading to the activation of downstream signaling pathways, such as the phosphoinositide 3-kinase-protein kinase B (PI3K-AKT) and mitogen-activated protein kinase (MAPK) pathways, which promote cell survival and proliferation.^2^ Given the critical role of HER2-HER3 signaling in *NRG1* fusion-positive tumors, targeting these receptors represents a promising therapeutic strategy. Zenocutuzumab (MCLA-128), marketed as *Bizengri*, is a fully humanized bispecific antibody targeting HER2 and HER3. It operates through a unique “dock and block” mechanism: one arm of the antibody binds to HER2, while the other binds to HER3, thereby preventing the interaction between NRG1 and HER3, and inhibiting HER2-HER3 heterodimerization (Fig. 1a). Of note, preclinical studies have demonstrated that zenocutuzumab can significantly inhibit *NRG1* fusion-positive tumor cell growth in vitro and in vivo.^3^

In the eNRGy trial, patients with confirmed *NRG1* fusion-positive solid tumors, identified via next-generation sequencing (DNA- or RNA-based), were treated with zenocutuzumab. A total of 204 patients were enrolled, of whom 158 had measurable disease and were followed for at least 24 weeks prior to the data-cutoff date. These patients represented 10 different tumor types (Fig. 1b), with the majority being NSCLC (58%) and *KRAS* wild-type pancreatic cancer (22%). The median age of participants was 62 years (range: 21 to 88 years), and the majority were female (60%). The most common *NRG1* fusion partners identified were *CD74* (35%), *SLC3A2* (14%), and *ATP1B1* (11%).

The reported objective response rate (ORR) was 30% (95% confidence interval [CI], 23–37%), with a median duration of response of 11.1 months (95% CI, 7.4–12.9 months). The median progression-free survival (PFS) was 6.8 months (95% CI, 5.5–9.1 months). Tumor regression was observed in 72% of the participants across multiple tumor types and *NRG1* fusion partners, underscoring significant antitumor activity. Notably, subpopulation analysis revealed that patients with NSCLC achieved an ORR of 29% and a PFS of 6.8 months (95% CI, 5.3–7.5 months), while patients with pancreatic cancer exhibited a remarkable ORR of 42% and a median PFS of 9.2 months (95% CI, 5.5–11.2 months). Although these findings pertain to a highly selected patient population, they are particularly encouraging for pancreatic cancer, a historically difficult-to-treat tumor with limited therapeutic options. Treatment-related adverse events (TRAEs) were reported in 95% of participants, with fewer than 10% being grade 3 or higher. Notably, cardiac toxicities, a known risk associated with wild-type HER2, were only observed in a small number of cases and had no clinical impact on patient outcomes.

These findings are especially notable considering that 91% of trial participants had received prior systemic therapies, including chemotherapy, immunotherapy, hormonal agents, and targeted treatments. Notably, a subset of patients had previously received treatment with afatinib, a pan-ERBB inhibitor. Interestingly, recent results from the Phase 2 TAPUR basket study (NCT02693535) showed that afatinib exhibited clinical activity in three patients with *NRG1* fusion-positive tumors, including lung, colorectal, and pancreatic tumor types (Fig. 1c).^4^ However, larger cohort studies are needed to confirm its efficacy in *NRG1* fusion-positive tumors. Furthermore, since NRG1 fusion proteins act as ligands for HER3, this has prompted the development of therapies that target HER3 directly. Several such agents are currently under investigation, including monoclonal antibodies, such as seribantumab (NCT04383210), GSK2849330 (NCT01966445), and HMBD-001 (NCT05057013), as well as antibody-drug conjugates, such as patritumab deruxtecan (NCT06383884). Early data from the CRESTONE trial reported an ORR of 36% with seribantumab in patients with *NRG1* fusion-positive solid tumors.^5^ Nonetheless, the compelling efficacy and safety profile of zenocutuzumab supported its accelerated FDA approval, making it the first approved targeted therapy for *NRG1*-positive NSCLC and pancreatic adenocarcinoma.

Although the clinical response to zenocutuzumab has been highly encouraging, further research is needed to fully understand and strengthen its therapeutic potential. First, while the observed ORR of 30% and median duration of response of 11.1 months are promising, the mechanisms driving primary and secondary resistance remain poorly understood. Elucidating these pathways could inform the development of rational combination therapies, extending clinical benefits to a larger patient population. Second, although *NRG1* fusions are found in multiple tumor types, zenocutuzumab has only received FDA approval for NSCLC and pancreatic adenocarcinoma, unlike tumor-agnostic agents such as larotrectinib and entrectinib (for *NTRK* fusion-positive tumors) or selpercatinib (for *RET* fusion-positive tumors). Zenocutuzumab has also shown activity in cholangiocarcinoma, breast cancer, and other tumor types. However, the limited number of patients with these cancers in this trial makes it difficult to draw definitive conclusions. Expanded studies with larger cohorts are needed to further evaluate its efficacy across other tumor types. Finally, reliable detection of the *NRG1* fusion, a critical biomarker for patient selection, remains a key challenge, particularly with non-invasive liquid biopsy approaches. Advancements in diagnostic technologies, such as nanopore sequencing, could enhance the accuracy of fusion detection, enabling better patient stratification and dynamic monitoring of fusion expression during treatment.

In summary, this phase II study demonstrated the clinically meaningful efficacy of zenocutuzumab in *NRG1* fusion-positive tumors with a favorable safety profile. These results establish *NRG1* fusions as an actionable therapeutic target and support the further development of novel therapeutic strategies targeting these fusions. It further highlights the crucial role of molecular profiling in identifying rare yet clinically significant targetable alterations.
